# KRT16 and APOA1: key regulators of proliferation and lipid metabolism in non-small cell lung cancer

**DOI:** 10.1515/biol-2025-1291

**Published:** 2026-04-17

**Authors:** MeiE Yu, Ling Zhu, YingXiao Wu, MengLing Li, QiongYing Wei

**Affiliations:** Department of Pulmonary and Critical Care Medicine, Fujian Medical University Union Hospital, Fuzhou, Fujian, 350001, China

**Keywords:** KRT16, APOA1, non-small cell lung cancer, lipid droplet accumulation, tumor progression

## Abstract

This study aimed to analyze the biological function of KRT16 and its interaction mechanism with apolipoprotein A1 (APOA1) in Non-Small Cell Lung Cancer (NSCLC). KRT16 expression and prognostic value in NSCLC were analyzed using the TCGA, GEPIA, and STRING databases. Functional impacts were assessed in A549 cells transfected with shKRT16 or APOA1 overexpression vectors. Gene/protein expression (RT-qPCR, Western blot), proliferation (MTT, EdU), cell cycle/apoptosis (flow cytometry), migration/invasion (Transwell), and lipid droplet accumulation (Oil Red O, Nile Red staining) were evaluated. The KRT16-APOA1 interaction was verified by co-immunoprecipitation, immunofluorescence, and GST pull-down assays. In NSCLC, KRT16 was up-regulated, and its high levels were associated with advanced tumor stage, lymph node metastasis, and poor prognosis. KRT16 levels were significantly elevated in patients aged 21–40 and were associated with *Helicobacter pylori* infection. Knockdown of KRT16 or overexpression of APOA1 inhibited proliferation, induced cell cycle arrest, promoted apoptosis, and reduced the accumulation of lipid droplet in A549 cells. KRT16 promoted NSCLC cell proliferation, migration, and lipid droplet accumulation by directly interacting with APOA1. KRT16 is highly expressed in NSCLC and regulates the malignant behaviors of NSCLC cells by interacting with APOA1.

## 1 Introduction

Lung cancer, known for its high morbidity and mortality rates worldwide, is histopathologically divided into non-small cell lung cancer (NSCLC) and small-cell lung cancer [1]. NSCLC constitutes the predominant subtype, accounting for approximately 85 % of cases [2], 3]. Although advancements in surgical, radiotherapy, chemotherapy, targeted therapy, and immunotherapy have improved survival outcomes of NSCLC patients, the overall treatment outlook remains suboptimal due to therapeutic limitations, high recurrence rates, poor prognosis, and low survival rates [4], [5], [6]. A major challenge in improving cure rates is cancer immune escape [7]. Several factors contribute to immune escape in tumor cells, including missing co-stimulatory antigens, reduced killer immune cells, increased number of immunosuppressive cells, and increased immunosuppressive factors in the tumor microenvironment [8], 9]. Furthermore, increasing evidence indicates that metabolic reprogramming plays a crucial role in tumor progression and the establishment of an immunosuppressive tumor microenvironment in both non-small cell lung cancer and small cell lung cancer. Therefore, it is essential to investigate the immune escape mechanism in NSCLC and identify new therapeutic targets.

Targeting energy metabolism to prevent the malignant phenotype of tumor cells is an emerging strategy in cancer treatment [10]. Metabolic changes in cancer cells are significantly different from those in normal cells [11]. Notably, cancer cells exhibit uncontrolled growth even under nutrient-deficient conditions. In particular, lipid metabolic reprogramming – characterized by enhanced *de novo* fatty acid synthesis, increased lipid droplet accumulation, and dysregulated cholesterol transport – plays a pivotal role in supporting NSCLC progression. In cancer cells, lipid metabolism undergoes significant changes, with an increase in *de novo* fatty acid synthesis, unlike normal cells which mainly rely on exogenous fatty acid uptake. This process is crucial for cell membrane synthesis and signaling [12]. Fatty acid synthesis begins with citric acid, with ATP citrate lyase converting it into acetyl coenzyme A. This is then changed into malonyl coenzyme A by acetyl coenzyme A carboxylase 1, and fatty acids are subsequently synthesized via fatty acid synthase [13].

Keratin 16 (KRT16), a member of the keratin family, is significantly up-regulated during the differentiation of complex epithelial cells. Other keratins in this family, such as KRT6, KRT17, and KRT19, have already been implicated in lung cancer progression: KRT6 promotes NSCLC cell invasion by regulating matrix metalloproteinases, KRT17 correlates with lymph node metastasis, and KRT19 serves as a diagnostic marker for minimal residual disease, collectively underscoring the family’s significant role in epithelial tumorigenesis. KRT16 dysfunction has been associated with inflammatory reactions, epidermal stress, and the cancerous transformation of epithelial-derived tumors [14], 15]. Elevated expression of KRT16 has been detected in multiple tumors and is closely related to tumor growth, invasion, and negative prognosis [16], 17]. A study by Li et al. identified KRT16 as a key gene in lipid metabolism; knockdown of KRT16 suppressed the proliferation, migration, and invasion of CCA cells [18]. However, the mechanism of action in NSCLC is largely unexamined, with its involvement in lipid metabolism regulation still unreported.

In recent years, the role of lipid metabolism in cancer has been extensively explored. Tumor cells reprogram lipid metabolism to meet their needs for growth and metastasis [19]. Specifically, alterations in lipid metabolism can promote tumor cell growth, invasion, and metastasis, as well as regulate the characteristics of the tumor microenvironment through intracellular signaling pathways [20]. Apolipoprotein A1 (APOA1), a common index of blood lipids, is believed to play an essential role in the occurrence and progression of cardiovascular diseases [21]. Recently, APOA1 has been associated with survival in patients with colorectal [22] and ovarian [23]. Functionally, APOA1 is a significant structural protein in HDL, contributing to lipid balance by promoting cholesterol efflux and preventing lipid peroxidation. For instance, studies have shown that APOA1 encoding recombinant adenovirus remodels cholesterol metabolism in tumors and the tumor microenvironment to inhibit hepatocellular carcinoma [24].

While APOA1 is well-researched in cardiovascular diseases, its role in the abnormal lipid metabolism of NSCLC is not yet fully understood.

Given the importance of abnormal lipid metabolism in NSCLC progression and the potential connection between KRT16 and APOA1 in tumor biology, this study focuses on elucidating the role of the KRT16-APOA1 axis in lipid metabolism in NSCLC.

## 2 Materials and methods

### 2.1 Cell culture

Human NSCLC cell line A549 (National Collection of Authenticated Cell Cultures, China) was maintained in DMEM or RPMI-1640 (Gibco, USA) supplemented with 10 % fetal bovine serum (Gibco, USA) and 1 % penicillin-streptomycin (Yeasen, China). The culture was maintained at 37 °C and 5 % CO_2_.

### 2.2 Cell transfection

A549 cells cultured to 80 % confluence (2 × 10^5^ cells/well in 6-well plates) were transfected with pcDNA3.1 expressing green fluorescence (FITC) carrying APOA1 target gene or empty vector (Sangon, China) using Lipofectamine 3000 (Invitrogen, USA). The culture medium was replaced after 6 h. To establish stably overexpressing cell lines, transfected cells were screened with 2 μg/ml puromycin (Thermo Fisher Scientific, USA) for 72 h. GFP expression was confirmed using a fluorescence microscope (DMi1; Leica, USA). Screened cells were subjected to stable passaging to produce A549 cells that consistently overexpress APOA1, with their overexpression efficiency evaluated by RT-qPCR and Western blot. Using Lipofectamine 3000 (Invitrogen), small interfering RNAs (sh-KRT16, sh-APOA1) that specifically bind to KRT16 and APOA1, as well as shCtrl, were transfected into A549 cells at 50 nM, with the medium being changed 6 h post-transfection. After 48 h of transfection, cells were gathered to measure transfection efficiency using RT-qPCR and Western blot. All sh-RNAs were synthesized by RiboBio (China). Lentiviral plasmids were purchased from GENE company.

### 2.3 RT-qPCR

Total cellular RNA was extracted using TRIZOL reagents (Thermo Fisher Scientific) and synthesized into cDNA using the TransScript All-in-One First-Strand cDNA Synthesis SuperMix for RT-PCR kit (TransGen, Beijing, China). qPCR was performed on a Step-One Plus Real-Time Fluorescent Quantitative PCR System (Thermo Fisher Scientific) using qPCR SYBR Green premix (Vazyme, China). The relative mRNA expression was calculated by the 2^−ΔΔCT^ method, with GAPDH as the internal reference. The primer sequences used were as follows: KRT16 (Forward: 5′-GAC​CGG​CGG​AGA​TGT​GAA​C-3′; Reverse: 5′-CTG​CTC​GTA​CTG​GTC​ACG​C-3′), APOA1 (Forward: 5′-CTA​AAG​CTC​CTT​GAC​AAC​TGG​G-3′; Reverse: 5′-TTT​CCA​GGT​TAT​CCC​AGA​ACT​C-3′), GAPDH (Forward: 5′-GTC​TCC​TCT​GAC​TTC​AAC​AGC​G-3′, Reverse: 5′-ACC​ACC​CTG​TTG​CTG​TAG​CCA​A-3′).

### 2.4 Western blot

Cells were lysed in RIPA buffer (Beyotime, China) and incubated on ice for 20 min. Protein concentration was determined usingh the Bradford assay (Bio-Rad, USA). Proteins were then separated by 15 % SDS-PAGE, electrophoretically transferred to a PVDF membrane, and blocked with 5 % skim milk powder for 1 h at room temperature. The membranes were incubated overnight at 4 °C with the following primary antibodies against KRT16 (ab76416, Abcam, USA), caspase-3 (ab32042), Bax (ab32503), Bcl-2 (ab182858), Fas (ab133619), p21 (ab109520), cyclin D1 (ab16666), and p53 (ab26), all sourced from Abcam, were incubated overnight at 4 °C. Subsequently, the membrane was incubated with horseradish peroxidase-conjugated secondary antibodies (CST, USA) at 37 °C for 1 h. Protein bands were visualized using the Enhanced Chemiluminescence Kit (ultrassignal, China). GAPDH served as the loading control.

### 2.5 MTT assay

A549 cells (5 × 10^3^/well in 96-well plates) were incubated for 24, 48, 72, and 96 h. At each interval, 20 μl of MTT solution at 5 mg/ml (Sigma, USA) was added to each well and incubated at 37 °C for 4 h. The formazan crystals were then dissolved by adding 100 µl of dimethyl sulfoxide to each well. Absorbance was measured at 570 nm using a microplate reader.

### 2.6 Oil red O staining

A549 cells were fixed with 4 % paraformaldehyde for 15 min, rinsed with 60 % isopropyl alcohol for 20–30 s, and then stained with Oil Red O solution for 15 min in the dark. After staining, cells were rinsed again with 60 % isopropyl alcohol and counterstained with Mayer’s hematoxylin solution for 3 min. Images were captured under a light microscope. The number of lipid droplet-positive cells was quantified in at least five randomly selected fields per sample.

### 2.7 Nile red staining

After washing with PBS, cells were fixed with 4 % paraformaldehyde for 15 min, then incubated with Nile Red staining solution for 20 min in the dark, followed by DAPI staining for 10 min (all steps protected from light). Images were captured using a fluorescence microscope, and the number of Nile Red-positive cells was counted in at least five randomly selected fields per sample.

### 2.8 Immunofluorescence assay

Cells were seeded in 24-well plates at 3 × 10^4^ cells per well and cultured overnight. After fixation with 4 % paraformaldehyde and permeabilization with 0.5 % Triton X-100 for 15 min, cells were blocked with 1 % BSA for 2 h at room temperature. Subsequently, samples were incubated overnight at 4 °C with primary antibodies against KRT16 (1:500, ab76416, Abcam) or APOA1 (1:500, ab52945, Abcam). After washing, cells were incubated for 1 h at room temperature in the dark with fluorescently conjugated secondary antibodies: goat anti-rabbit IgG (1:500, ab6939, Abcam) and goat anti-mouse IgG (1:500, ab97035, Abcam). Nuclei were stained with DAPI. Images were acquired by fluorescence microscopy (Olympus, Japan).

### 2.9 Co-immunoprecipitation (CO-IP)

Cells were lysed with RIPA lysis buffer and centrifuged at 4 °C to collect the supernatant. The supernatant was first incubated with Protein A/G Magnetic Beads (#sc-2003, Santa Cruz, USA) for 2 h at 4 °C to pre-clear nonspecific binding. Then, 500 μg of protein lysate was incubated overnight at 4 °C with anti-KRT16 antibody (1:500, ab76416, Abcam) or control IgG (1:500, #AC005, ABclonal, USA), followed by the addition of fresh Protein A/G Magnetic Beads for an additional 2 h. After washing three times with cold RIPA buffer, bound proteins were eluted by boiling in 1 × SDS loading buffer and analyzed by Western blot using 20 μg of protein per sample.

### 2.10 Flow cytometry

Cell apoptosis rate was assessed by Fluorescein Isothiocyanate (FITC) Annexin V Apoptosis Detection Kit (BD Biosciences, USA). After 48 h of transfection, A549 cells were resuspended in 500 μl of 1 × binding buffer and stained with 5 μl Annexin V-FITC and 5 μl propidium iodide (PI) for 15 min at room temperature in the dark. The apoptotic cell population was analyzed using a FACScan flow cytometer (BD Biosciences).

For cell cycle analysis, cells were fixed overnight in ice-cold 70 % ethanol at 4 °C, washed with PBS, and then stained with 20 μg/ml PI containing RNase A for 30 min in the dark. DNA content was analyzed using a flow cytometer (BD Bioscience).

### 2.11 Transwell assay

Transwell chambers (BD Biosciences) precoated with Matrigel (BD Biosciences) to measure cellular invasion, while uncoated chambers were used for migration assays. A549 cells were collected 48 h after transfection and resuspended in FBS-free DMEM to 5 × 10^6^ cells/ml. Next, 200 μl of cell suspension was added to the upper compartment, and 500 μl of DMEM +20 % FBS was added to the lower compartment. After 24 h, cells were fixed with 4 % paraformaldehyde and then stained with 0.5 % crystal violet. Five randomly selected fields per membrane were imaged under an inverted microscope (Olympus, Japan), and stained cells were counted.

### 2.12 Scratch test

Cells were cultured in 6-well plates at 2.5 × 10^4^ cells/cm^2^. After 24 h, a scratch wound was created using a sterile 10 μL pipette tip. Cells were then incubated in RPMI 1640 medium containing 10 % FBS. Wound healing was observed and recorded at 0 h and 48 h.

### 2.13 Data analysis

All statistical analyses were performed using GraphPad Prism6. Data are presented as mean ± standard deviation (SD). Comparisons between two groups were analyzed using the unpaired Student’s *t*-test, while comparisons among multiple groups were performed by one-way ANOVA followed by an appropriate post-hoc test. Survival analysis was conducted using the Kaplan–Meier method and compared with the log-rank test. A *P* values < 0.05 were considered statistically significant.

## 3 Results

### 3.1 KRT16 expression signature in lung cancer

**Figure 1:**
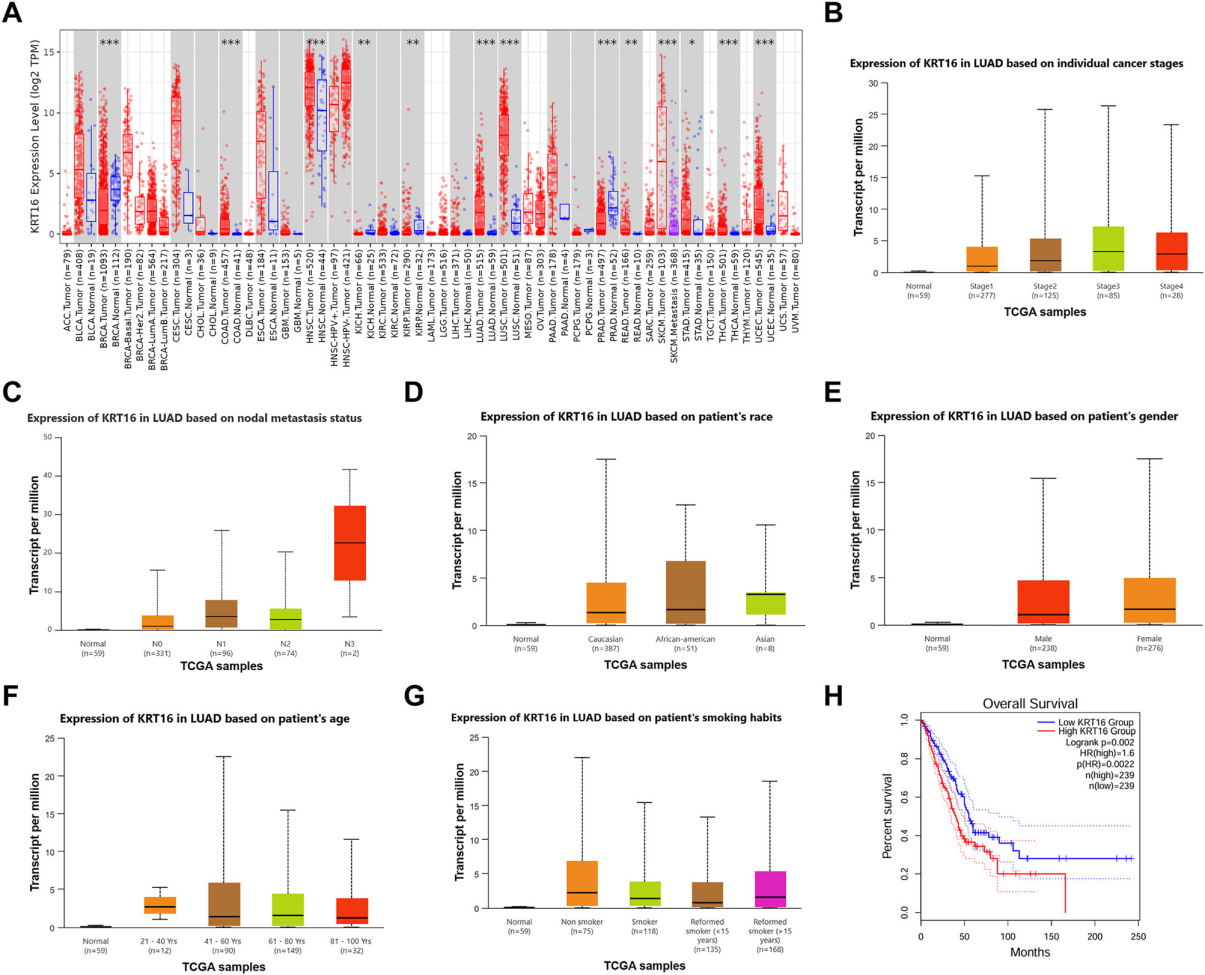
KRT16 is upregulated in lung cancer and correlates with poor prognosis. (A) Expression of KRT16 in normal and lung cancer tissues in the TCGA dataset. (B) Expression of KRT16 in lung cancer patients with different tumor stages in the TCGA dataset. (C) Expression of KRT16 in lung cancer tumor tissues of different metastatic stages in the TCGA dataset. (D) Expression of KRT16 in lung cancer tumor tissues of different races in the TCGA dataset. (E) Expression of KRT16 in lung cancer tumor tissues of different genders in the TCGA dataset. (F) Expression of KRT16 in lung cancer tumor tissues of different ages in the TCGA dataset. (G) Expression of KRT16 in lung cancer tumor tissues from smoking and non-smoking lung cancer patients. (H) Survival of KRT16 in lung cancer patients in the TCGA dataset.

Analysis of lung cancer-related datasets from the TCGA database revealed that KRT16 was significantly upregulated in lung cancer tissues (Figure 1A). Elevated KRT16 expression was associated with primary tumors, advanced tumor stage (stage II-IV), and lymph node metastasis (N stage) (Figure 1B and C). KRT16 expression was also significantly increased in Asian patients with lung cancer (Figure 1D). Compared with normal individuals, KRT16 levels were higher in both male and female lung cancer patients, though no significant gender-based differences was observed (Figure 1E). In patients aged 21–40 years, KRT16 expression in tumor tissues was markedly elevated relative to that in age-matched healthy controls (Figure 1F). There was no significant difference in KRT16 expression between smokers and non-smokers compared to normal patients (Figure 1G). Survival analysis performed on the GEPIA website showed that lung cancer patients with higher KRT16 expression had significantly poorer overall survival (*P* = 0.00024) and recurrence-free survival (*P* = 0.00016) (Figure 1H).

### 3.2 KRT16 knockdown inhibits proliferation and reduces lipid droplet accumulation in NSCLC A549 cells

**Figure 2:**
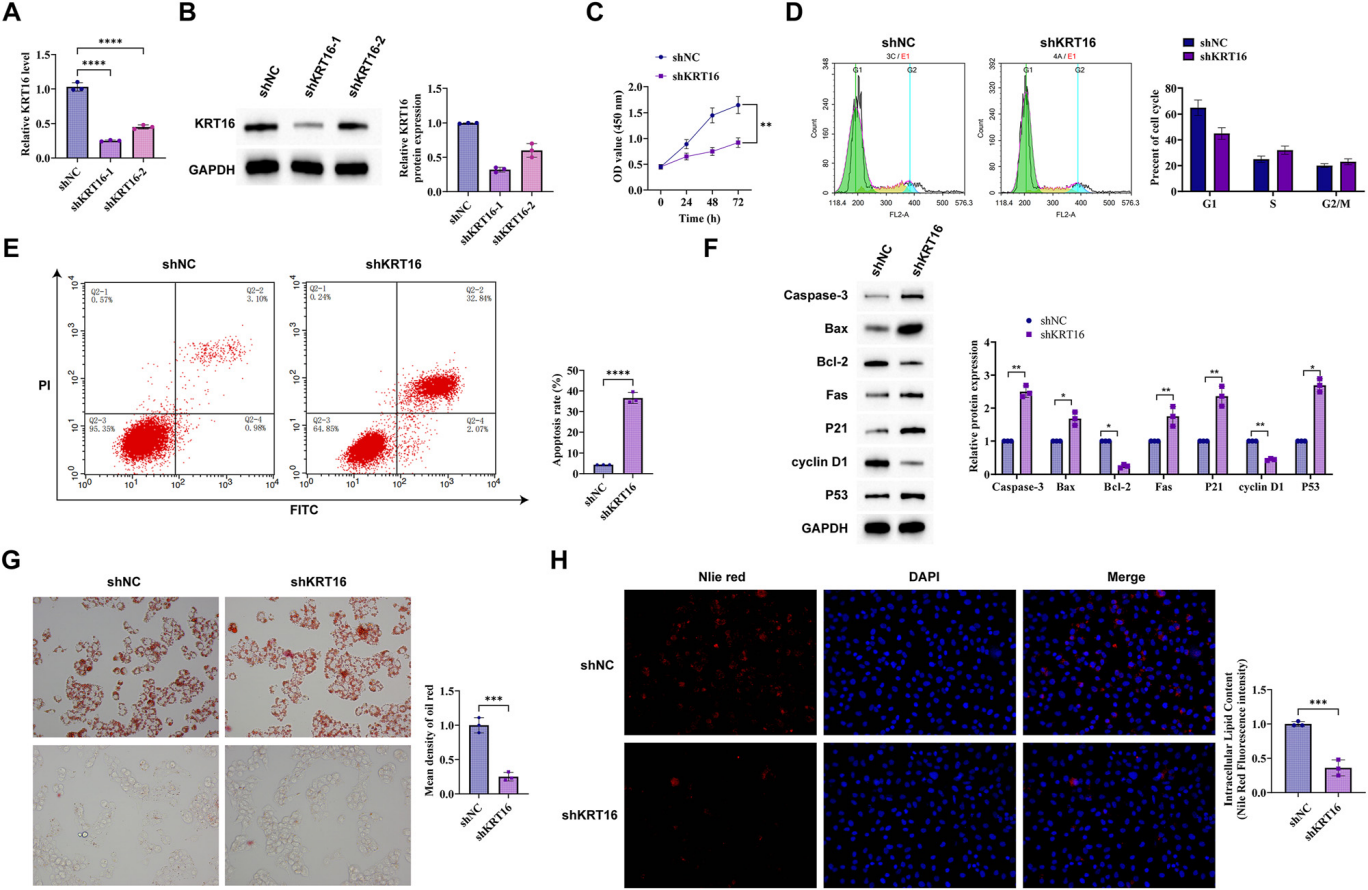
KRT16 knockdown inhibits proliferation and reduces lipid droplet accumulation in A549 cells. (A) RT-qPCR to detect KRT16 mRNA expression after shCtrl and shKRT16 transfection of A549 cells. (B) Western blot to detect KRT16 protein expression after shCtrl and shKRT16 transfection of A549 cells. (C) MTT assay to determine the effect of shNC or shKRT16 on cell proliferation. (D) Flow cytometry assay to detect the effect of shNC or shKRT16 on cell cycle. (E) Flow cytometry assay to detect the effect of shNC or shKRT16 on cell apoptosis. (F) Western blot to detect KRT16, caspase-3, bax, Bcl-2, Fas, p21, cyclin D1 and p53. (G) Oil red O staining to detect the effect of KRT16 on lipid droplet accumulation in lung cancer cells (400 × ). (H) Nile red staining to detect the effect of KRT16 on lipid droplet accumulation in lung cancer cells (400 × ) the data are expressed as the mean ± SD of three independent experiments. Each group of experiments was independently repeated three times. *P < 0.05, **P < 0.01, ***P < 0.001, ns, not significant.

To investigate the role of KRT16 in cancer progression, A549 cell lines were successfully transfected with specific shRNA lentiviral vectors (shKRT16) and negative control vectors (shNC) targeting KRT16. RT-qPCR and Western blot assays showed that KRT16 expression was significantly reduced after shKRT16 treatment (Figure 2A and B). MTT assay confirmed that KRT16 knockdown suppressed A549 cell proliferation (Figure 2C). Flow cytometry analysis showed that KRT16 knockdown significantly decreased the proportion of cells in S phase and increased the percentage of early apoptotic cells (Figure 2D and E). Western blot analysis indicated that pro-apoptotic proteins (cleaved caspase-3, Bax, and Fas) and cell cycle regulators (p21 and p53) were upregulated, while anti-apoptotic protein Bcl-2 and cell cycle protein cyclin D1 were downregulated after KRT16 knockdown (Figure 2F). In terms of lipid metabolism, Oil Red O staining showed that shNC-treated cells contained numerous red lipid droplets of varying sizes and irregular distribution, whereas shKRT16 treatment led to a marked reduction in lipid droplets (Figure 2G). Consistent with this, Nile Red staining demonstrated significantly decreased lipid fluorescence intensity following KRT16 knockdown (Figure 2H).

### 3.3 Knockdown of APOA1 promotes cell proliferation and lipid droplet accumulation in NSCLC cells

**Figure 3:**
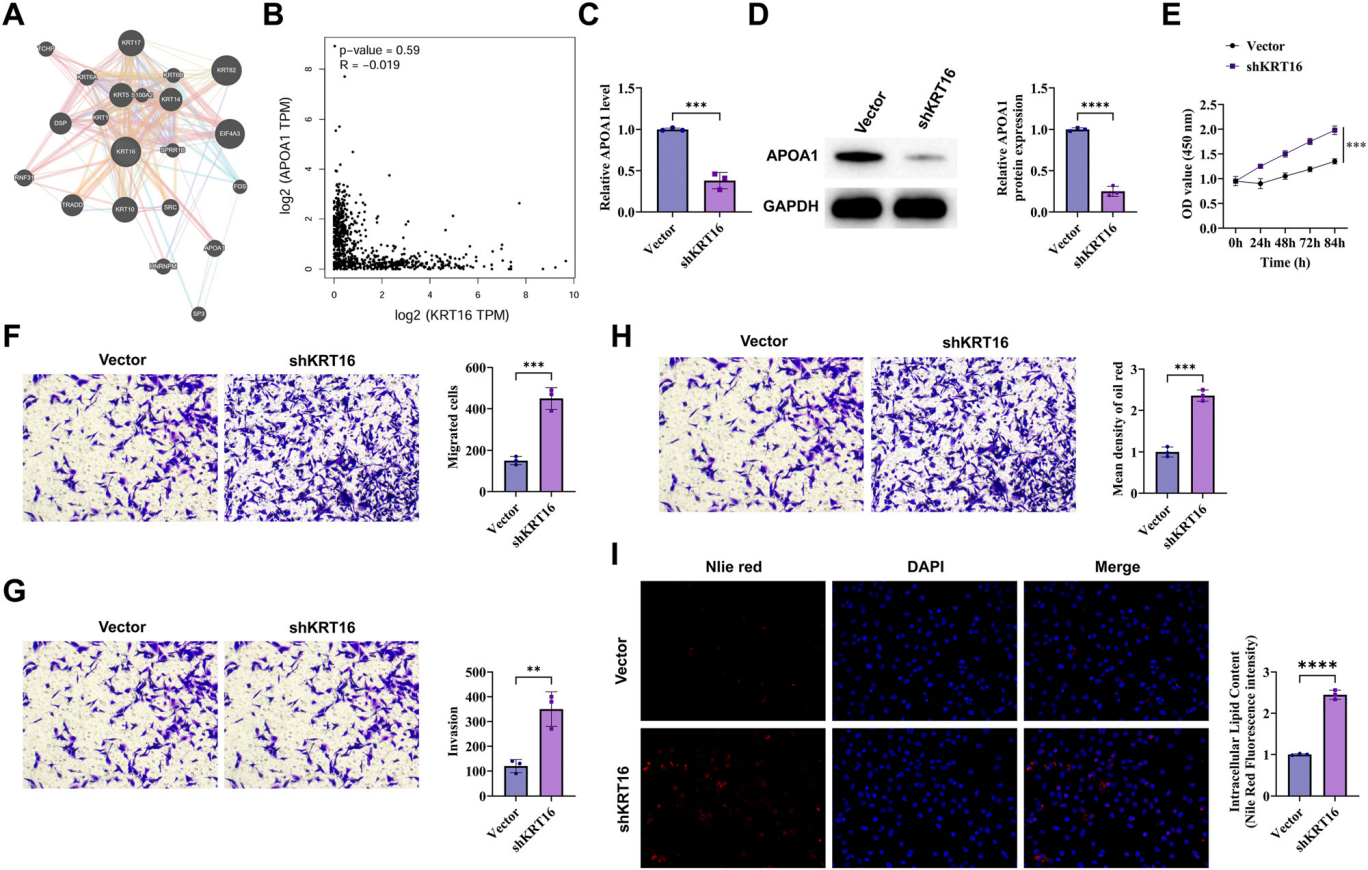
Overexpression of APOA1 inhibits proliferation and reduces lipid droplet accumulation in NSCLC cells. (A) String to predict the binding site of KRT16 with APOA1. (B) GEPIA to predict the correlation between KRT16 and APOA1 in lung cancer. (C) RT-qPCR to detect mRNA expression of APOA1 after transfection of A549 cells with vector and shAPOA1. (D) Western blot to detect the protein expression of APOA1 after vector and shAPOA1 transfection of A549 cells. (E) MTT assay to determine the effect of vector or shAPOA1 on cell proliferation. (F) Transwell assay to detect the effect of vector or shAPOA1 on cell migration ability. (G) Transwell assay to detect the effect of vector or shAPOA1 on cell invasion ability. (H) Oil red O staining to detect the effect of vector or shAPOA1 on lipid droplet accumulation (400 × ). (I) Nile red staining to detect the effect of vector or shAPOA1 on lipid droplet accumulation cells (400 × ). The data are expressed as the mean ± SD of three independent experiments. Each group of experiments was independently repeated three times. *P < 0.05, **P < 0.01, ***P < 0.001, ns, not significant.

The STRING database predicted a potential interaction between KRT16 and APOA1 (Figure 3A). Analysis using GEPIA indicated that KRT16 expression was negatively correlated with APOA1 expression in lung cancer (Figure 3B). Specific shRNA lentiviral vector targeting APOA1 (shAPOA1) and a negative control vector (shNC) were constructed and successfully transfected into A549 cell line. RT-qPCR and Western blot assays showed that APOA1 expression was significantly reduced following shAPOA1 transfection (Figure 3C and D). The MTT assay revealed that knockdown of APOA1 enhanced the proliferation of A549 cells (Figure 3E). Transwell assays further showed that silencing APOA1 promoted the migration and invasion abilities of A549 cells (Figure 3F and G). Oil Red O staining showed red lipid droplets of varying sizes and irregular distribution in both control and shAPOA1-treated cells, with the shAPOA1 group displaying more abundant lipid droplets (Figure 3H). In addition, Nile Red staining demonstrated a significant increase in fluorescence intensity after APOA1 knockdown (Figure 3I).

### 3.4 Interaction between KRT16 and APOA1

**Figure 4:**
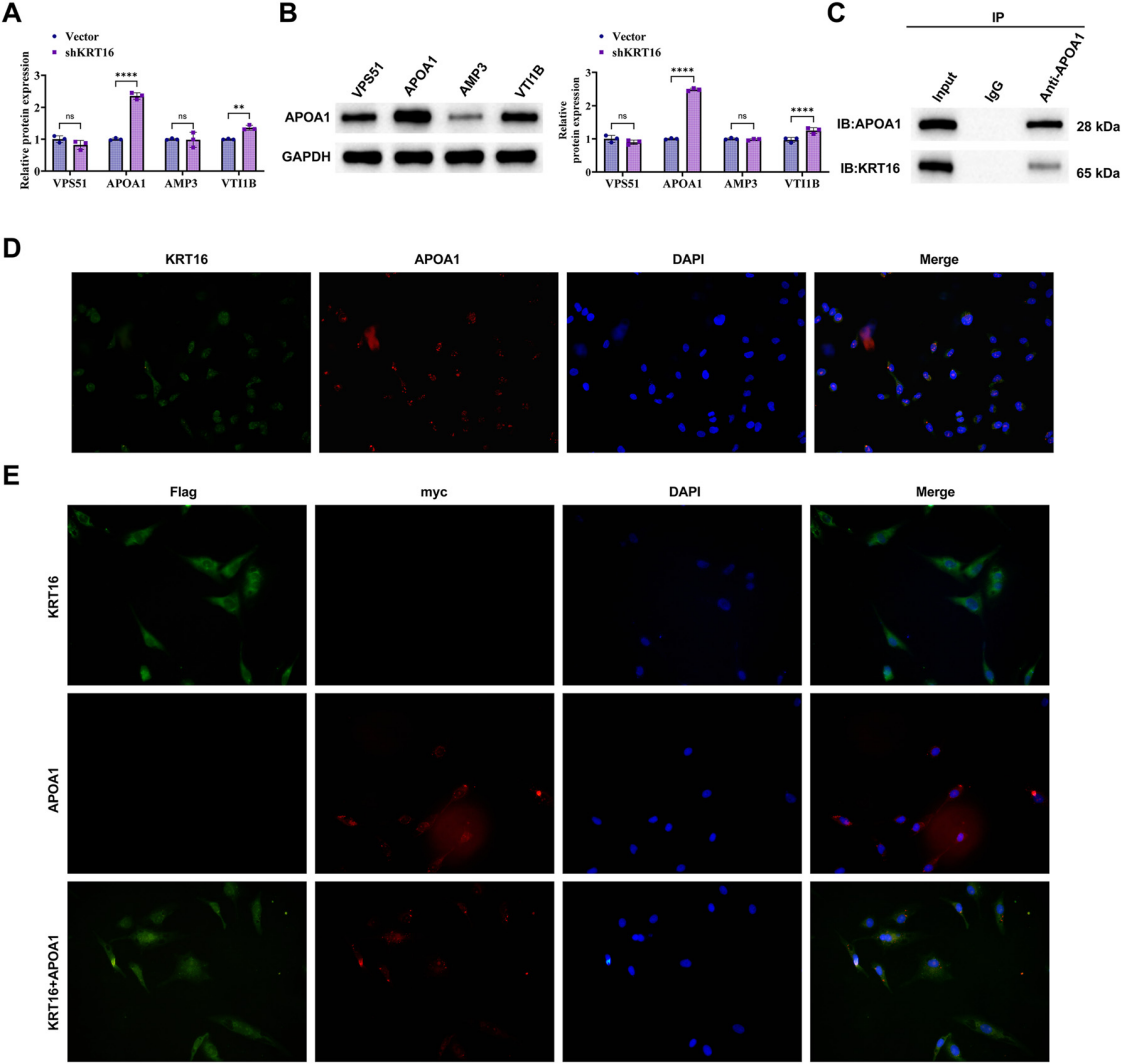
KRT16 interacts with APOA1. (A) RT-qPCR analysis of VPS51, VTI1B, APOA1, and VAMP3 mRNA expression. (B) Western blot to detect the expression levels of VPS51, VTI1B, APOA1, and VAMP3 protein. (C) CO-IP validation of the interaction between KRT16 and APOA1. (D) Immunofluorescence localization of KRT16 with APOA1. (E) Interaction between KRT16 and APOA1 in GST pull-down assay. The data are expressed as the mean ± SD of three independent experiments. Each group of experiments was independently repeated three times. *P < 0.05, **P < 0.01, ***P < 0.001, ns, not significant.

The relationship between KRT16 and APOA1 was first examined using RT-qPCR and Western blot. Silencing KRT16 significantly increased APOA1 expression (Figure 4A and B). The interaction between KRT16 and APOA1 was confirmed by Co-IP (Figure 4C). Immunofluorescence analysis revealed that both proteins were co-localized in the cytoplasm. This finding was further validated in HEK293T cells co-transfected with Flag-KRT16 and Myc-APOA1, which showed clear pairing of the two proteins (Figure 4D). GST pull-down assays were performed using an *Escherichia coli* expression system. Purified proteins were analyzed by Western blot, which indicated that KRT16 bound directly to GST-APOA1 but not to GST alone (Figure 4E).

### 3.5 APOA1 knockdown partially reverses the inhibitory effects of KRT16 knockdown on proliferation and migration in NSCLC cells

**Figure 5:**
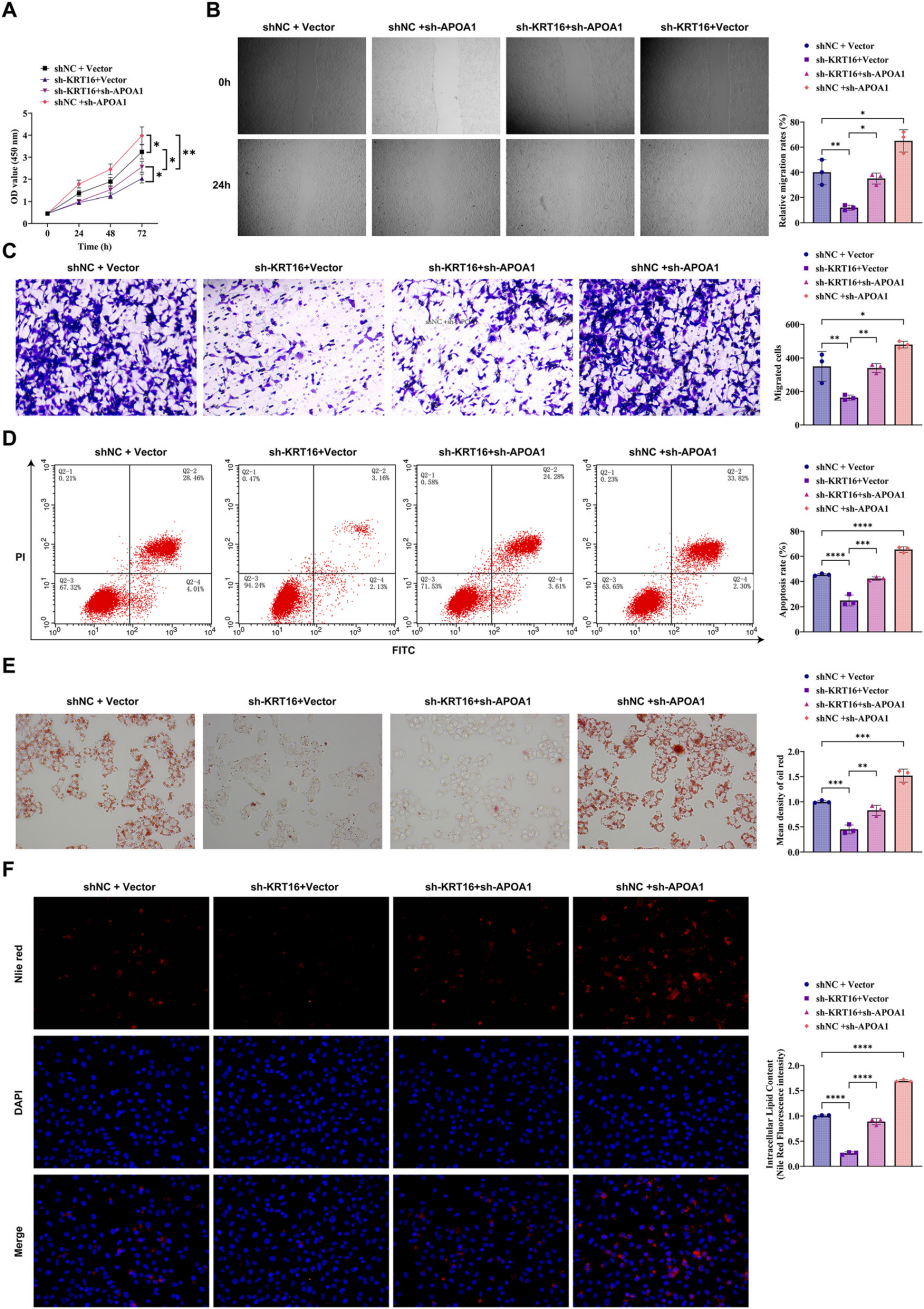
APOA1 knockdown partially reverses the inhibitory effects of KRT16 knockdown on proliferation and migration in NSCLC cells. (A) MTT assay for cell proliferation. (B) Scratch assay to detect cell migration ability. (C) Transwell assay to detect the effect of cell migration ability. (D) Flow cytometry to detect cell apoptosis. (E) Oil red O staining to detect lipid droplet accumulation (400 × ). (F) Nile red staining to detect lipid droplet accumulation cells (400 × ). The data are expressed as the mean ± SD of three independent experiments. Each group of experiments was independently repeated three times. *P < 0.05, **P < 0.01, ***P < 0.001, ns, not significant.

Data confirmed that both KRT16 and APOA1 independently regulate NSCLC progression. To determine whether they act synergistically, functional rescue experiments were performed. MTT and scratch wound-healing assays showed that, compared with the shNC + Vector group, the shKRT16 + Vector group exhibited a significant decrease in cell proliferation and migration, whereas the shKRT16 + shAPOA1 group showed increased proliferation and migration relative to the shKRT16 + Vector group (Figure 5A and B). Notably, the shNC + shAPOA1 group displayed lower proliferation and migration than the shNC + Vector group (Figure 5C). Flow cytometry analysis indicated that compared to the shNC + Vector group, the number of apoptotic cells went up in the sh-KRT16 + Vector group, and the sh-KRT16 + sh-APOA1 group had more apoptotic cells than the sh-KRT16 + Vector group. However, the number of apoptotic cells in the shNC + sh-APOA1 group was lower (Figure 5D). Oil Red O staining showed that compared to the shNC + Vector group, the sh-KRT16 + Vector group had less red lipid droplets, while the sh-KRT16 + sh-APOA1 group exhibited a large number of red lipid droplets compared to the sh-KRT16 + Vector group, but the number of red lipid droplets in the shNC + sh-APOA1 group was relatively low (Figure 5E). Nile Red experiments further confirmed that compared to the shNC + Vector group, the fluorescence intensity in the sh-KRT16 + Vector group dropped, and the fluorescence intensity in the sh-KRT16 + sh-APOA1 group was also enhanced compared to the sh-KRT16 + Vector group, but weakened compared to the shNC + sh-APOA1 group (Figure 5F). Collectively, these results demonstrate that KRT16 promotes lipid droplet accumulation in NSCLC cells by targeting APOA1, thereby enhancing cell proliferation, migration, and invasion.

## 4 Discussion

Lung cancer, one of the most common malignant tumors worldwide in terms of incidence and mortality, has complex pathological mechanisms and high heterogeneity. NSCLC accounts for 80 %–85 % of all lung cancer cases. Currently, clinical treatment still faces serious challenges such as rapid tumor progression and poor prognosis [17], 24]. Identifying key molecular targets involved in the development and progression of lung cancer and analyzing their regulatory networks are of great importance for developing novel diagnostic markers and therapeutic strategies [25], 26]. KRT16, a member of the cytoskeletal intermediate filament protein family, has been mainly studied in the past for its role in skin diseases. In recent years, it has been found to be abnormally expressed in various tumors and involved in the regulation of malignant phenotypes. APOA1, the main component of HDL, not only participates in lipid metabolism regulation but has also been confirmed to possess anti-tumor activity. However, its role in lung cancer and its relationship with KRT16 remain unclear. This study systematically explored the expression characteristics and biological functions of KRT16 in lung cancer, as well as its interaction mechanism with APOA1, through bioinformatics analysis and *in vitro* cell experiments, providing new experimental evidence for a deeper understanding of the pathogenesis of lung cancer.

Bioinformatics analysis serves as an important tool for screening differentially expressed genes associated with tumors. The TCGA database, as one of the largest cancer genomics databases, provides abundant resources for identifying tumor biomarkers [27]. This study analyzed lung cancer-related datasets from the TCGA database and found that KRT16 was significantly upregulated in lung cancer tissues, which is consistent with previous reports in lung adenocarcinoma [15], suggesting that KRT16 may act as a potential oncogene in lung cancer development. Further analysis of clinicopathological parameters revealed that high KRT16 expression was closely associated with primary tumors, advanced tumor stage (stage II–IV), and lymph node metastasis (N stage), indicating that KRT16 expression increases with tumor progression and may be involved in the invasion and metastasis of lung cancer. Tumor stage is a key indicator for evaluating the prognosis of lung cancer patients, and the 5-year survival rate of advanced lung cancer patients is significantly reduced. The correlation between high KRT16 expression and advanced tumor characteristics suggests that KRT16 may serve as a molecular marker for assessing the progression of lung cancer.

In population characteristic analysis, this study found that KRT16 expression was significantly elevated in Asian lung cancer patients, which has important clinical relevance. The pathogenesis of lung cancer exhibits notable ethnic differences; the genetic mutation profiles and epidemiological characteristics of lung cancer in Asian populations differ significantly from those in Western populations. For example, the incidence of EGFR mutations is higher in Asian patients with lung adenocarcinoma [28]. The specific high expression of KRT16 in Asian patients may be related to ethnic genetic backgrounds or environmental exposure factors, suggesting that KRT16 could be a specific molecular target for Asian lung cancer patients, providing new insights for ethnicity-specific precision therapy. Additionally, the study found that KRT16 was highly expressed in both male and female lung cancer patients with no significant gender differences, indicating that its abnormal expression in lung cancer is not influenced by gender and has broad potential application value.

Age-stratified analysis showed that KRT16 levels were significantly higher in tumor tissues of young lung cancer patients (21–40 years old) compared to healthy individuals of the same age group. Young lung cancer patients often present with aggressive disease and poor prognosis, and their pathogenesis may differ from that of older patients, such as a higher likelihood of carrying germline mutations or being more significantly affected by environmental factors. The high expression of KRT16 in young lung cancer patients may be involved in the development of lung cancer in this population, providing new molecular mechanistic clues for explaining the malignant phenotype of young patients and offering a potential marker for early diagnosis. Notably, this study found no significant difference in KRT16 expression between smokers and non-smokers with lung cancer. Given that smoking is a major risk factor for lung cancer, this result suggests that the KRT16-mediated pathway of lung carcinogenesis may be independent of smoking-related pathways, providing a new target for the treatment of non-smoking lung cancer patients.

The gene KRT16 is known to regulate cell cycle in lung adenocarcinoma [29]. Interestingly, KRT16 shows significant overexpression in gastric cancer tissues and has a strong correlation with lymph node metastasis and poor pathological grading [30]. KRT16 is regarded as a potential gene for regulating NSCLC development since it prevents lung cancer cell proliferation and interrupts the cell cycle by modulating key proteins at cell cycle checkpoints [29], 31]. As a core component of cytoskeletal intermediate filaments, KRT16 not only maintains cellular structural integrity but also directly participates in regulating intracellular signal transduction, organelle positioning, and protein-protein interactions – key processes that link cytoskeletal dynamics to metabolic remodeling and cellular homeostasis. The current research delved into understanding KRT16’s specific mechanism in NSCLC, highlighting innovative insights into lipid metabolism regulation. Cancer evolves over time due to the accumulated effects of factors and genetic mutations on the organism [32]. In tumor malignancy, tumor cells bypass the body’s regulatory controls, leading to invasive spread, unlimited proliferation, while energy metabolism supplies ‘logistical support’ to these cells. The metabolic pattern in tumor cells, characterized by active glutamine metabolism, an overly active glycolytic pathway, and abnormal lipid metabolism, is referred as energy metabolism reprogramming [33]. Cytoskeletal proteins like KRT16 contribute to this reprogramming by modulating the stability, subcellular localization, or functional interaction of metabolic regulators such as APOA1: KRT16 directly binds to APOA1 in the cytoplasm, which may interfere with APOA1-mediated cholesterol efflux (via ABCA1) or lipid droplet turnover, thereby shifting the balance of lipid metabolism toward lipogenesis and supporting tumor cell survival. Lipids contribute to the energy supply for lipid components of biological membranes, regulate lipid molecular signaling, and facilitate malignant biological behaviors including tumor cell invasion, metastasis, and proliferation. Elevated expression and activity of enzymes involved in lipid metabolism have been detected in many tumor cells, promoting fatty acid synthesis in cancer cells [33]. Alterations in lipid content or composition serve as indicators of cancer aggressiveness [18]. The findings that KRT16 promotes NSCLC cell proliferation and lipid droplet accumulation by directly interacting with APOA1 add a new layer to the understanding of metabolic reprogramming in NSCLC, as this axis specifically modulates lipid homeostasis – a key hallmark of the altered metabolic landscape that supports tumor progression. In this study, specific knockdown of KRT16 in A549 cells significantly inhibited tumor cell proliferation. This effect was closely associated with blocking cell cycle progression in the S phase and inducing early apoptosis. Molecular mechanistic studies revealed that downregulation of KRT16 significantly upregulated pro-apoptotic proteins (cleaved caspase-3, Bax, Fas) and key negative cell cycle regulatory proteins (p21, p53), while suppressing the anti-apoptotic protein Bcl-2 and cyclin D1. Furthermore, reducing KRT16 expression significantly decreased the buildup of lipid droplets in cells, as consistently demonstrated by Oil Red O staining and Nile Red fluorescence staining experiments. The buildup of lipid droplets suggests that tumor cells have a lipid metabolism imbalance that leans towards lipogenesis rather than lipolysis, equipping them with stronger energy reserves and metabolic adaptability to maintain their malignant phenotype and adapt to the tumor environment [34], 35].

To establish the direct regulatory relationship and functional synergy between KRT16 and APOA1, rigorous molecular interactions and functional rescue experiments were designed. First, APOA1 was significantly upregulated at both mRNA and protein levels upon KRT16 silencing. Subsequently, Co-IP experiments directly confirmed the direct interaction between KRT16 and APOA1 proteins. Immunofluorescence staining further revealed their co-localization in the cytoplasm. Critically, GST pull-down assay established that KRT16 directly binds to GST-tagged APOA1 (GST-APOA1), but not to GST alone, confirming the existence of a specific, direct protein-protein binding between the two. Functional rescue experiments provided stronger phenotypic evidence, showing that overexpressing of APOA1 significantly counteracted the inhibitory effects caused by KRT16 knockdown on cell proliferation, migration, and lipid droplet accumulation. APOA1, the main component of HDL, binds to the ABCA1 transporter protein, promotes cellular cholesterol efflux, facilitates the formation of HDL particles, and helps maintain lipid droplet homeostasis. In contrast, knockdown of APOA1 attenuated the tumor-promoting phenotype induced by APOA1 overexpression alone. These findings confirm that APOA1 acts as a significant downstream effector mediating the oncogenic effects of KRT16.

This study has several limitations that warrant further investigation. First, the research primarily relied on the A549 cell line and bioinformatics analysis; it lacks validation in animal models to confirm the oncogenic role of the KRT16-APOA1 axis *in vivo*. While the *in vitro* findings and database analyses provide robust support for the proposed mechanism, the absence of *in vivo* data remains a gap to be addressed in future work. Second, the precise molecular mechanism by which APOA1 regulates lipid droplet homeostasis is not fully elucidated: which specific pathways – fatty acid uptake, triglyceride synthesis, cholesterol transport, or endoplasmic reticulum stress – are modulated by APOA1 to influence lipid droplet formation? Third, the factors driving higher KRT16 expression in Asian patients – whether genetic, epigenetic, or environmental – are not yet explained. Integrated multi-omics analyses are needed to explore the causes and implications of this ethnic disparity. Fourth, the clinical therapeutic potential of targeting the KRT16-APOA1 axis remains to be explored: systematic screening of small-molecule or peptide inhibitors that disrupt this interaction, and evaluation of their anti-tumor efficacy alone or in combination with existing therapies, would be valuable. The therapeutic potential of APOA1 agonists in lung cancer also merits investigation.

In addition to the above points, other limitations should be noted. The cellular experiments were conducted using only the A549 cell line. Given the heterogeneity among NSCLC subtypes, whether the KRT16-APOA1 axis functions consistently across different NSCLC models requires further validation. Moreover, the precise mechanism by which KRT16 suppresses APOA1 expression – whether at the transcriptional, translational, or post-translational level – was not thoroughly investigated. Finally, clinical validation is limited, as this study relied mainly on TCGA database analysis and lacked large-scale validation with clinical specimens. The correlation between KRT16 and APOA1 expression and its clinical significance need confirmation with patient samples.

Future research should focus on the following directions: First, expanding the range of cell lines to validate the KRT16-APOA1 axis in NSCLC cells of different subtypes and genetic backgrounds will help determine its universality. Second, establishing animal models such as xenografts or genetically engineered mice will help verify the roles of KRT16 and APOA1 in tumor growth and metastasis *in vivo*. Third, the molecular mechanism of KRT16-mediated suppression of APOA1 should be further investigated – for example, using chromatin immunoprecipitation to examine whether KRT16 binds to the APOA1 promoter, or ubiquitination assays to determine its effect on APOA1 protein stability. Fourth, large-scale clinical lung cancer specimens should be analyzed by immunohistochemistry and RT-qPCR to validate the expression correlation between KRT16 and APOA1 and clarify their clinical relevance. Fifth, therapeutic strategies targeting the KRT16-APOA1 axis, such as KRT16-specific inhibitors or APOA1 overexpression vectors, should be explored and their potential in lung cancer treatment evaluated.

In summary, this study reveals the abnormal expression patterns and biological functions of KRT16 in lung cancer and clarifies the role of the KRT16-APOA1 regulatory axis in NSCLC progression. These findings provide new molecular targets and experimental evidence for the diagnosis, prognosis assessment, and targeted therapy of lung cancer, holding significant scientific value and clinical potential.
